# Direct regioisomer analysis of crude reaction mixtures *via* molecular rotational resonance (MRR) spectroscopy†
†Electronic supplementary information (ESI) available: MRR spectra, calculations, comparison between experimental and theoretical, separation methods. See DOI: 10.1039/d0sc01853h


**DOI:** 10.1039/d0sc01853h

**Published:** 2020-06-08

**Authors:** Leo A. Joyce, Danielle M. Schultz, Edward C. Sherer, Justin L. Neill, Reilly E. Sonstrom, Brooks H. Pate

**Affiliations:** a Department of Process Research & Development , Merck & Co., Inc. , Rahway , NJ 07065 , USA . Email: danielle.schultz@merck.com; b Department of Computational and Structural Chemistry , Merck & Co., Inc. , Rahway , NJ 07065 , USA; c BrightSpec, Inc. , 770 Harris St., Suite 104b , Charlottesville , VA 22904 , USA . Email: justin.neill@brightspec.com; d Department of Chemistry , University of Virginia , McCormick Road , Charlottesville , VA 22904 , USA

## Abstract

Direct analyses of crude reaction mixtures have been carried out using molecular rotational resonance (MRR) spectroscopy. Two examples are presented, a demonstration application in photocatalytic CH-arylation as well as generation of an intermediate in a natural product synthesis. In both cases, the reaction can proceed at more than one site, leading to a mixture of regioisomers that can be challenging to distinguish. MRR structural parameters were calculated for the low lying conformers for the desired compounds, and then compared to the experimental spectra of the crude mixtures to confirm the presence of these species. Next, quantitation was performed by comparing experimentally measured line intensities with simulations based on computed values for the magnitude and direction of the molecular dipole moment of each species. This identification and quantification was performed without sample purification and without isolated standards of the compounds of interest. The values obtained for MRR quantitation were in good agreement with the chromatographic values. Finally, previously unknown impurities were discovered within the photocatalytic CH-arylation work. This paper demonstrates the utility of MRR as a reaction characterization tool to simplify analytical workflows.

## Abstract 2

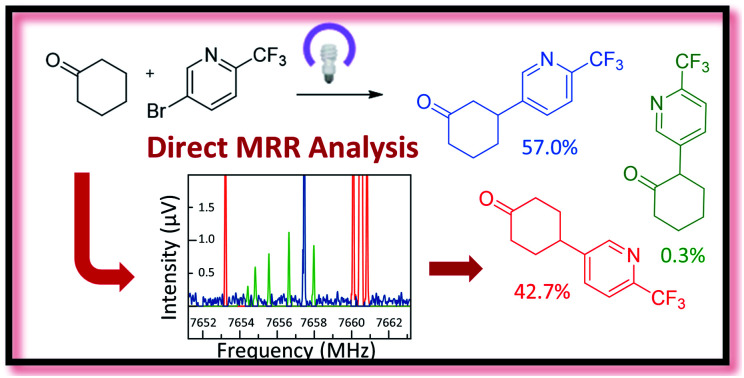
Direct analyses of crude reaction mixtures have been carried out using molecular rotational resonance (MRR) spectroscopy, allowing identification and quantification of major and minor components without sample purification or reference standards.

The characterization of novel synthetic chemical reactions – in particular, the identification and quantification of the products arising from a synthesis – is a critical, but often difficult task. Reactions often yield complex mixtures with structurally similar analytes, such as diastereomers, regioisomers, and positional isomers.^1^–^3^ The last few decades have seen the development of many synthetic platforms that give unique and exciting chemical transformations,^4^–^8^ so effective and standardized methods for reaction characterization are highly useful.

In order to fully understand the products of a reaction of interest, researchers often follow a procedure similar to the following. First, the reaction conditions are scaled up to generate a sufficient quantity of material for analysis. This may require running several concurrent reactions with identical conditions and pooling them together. Next, the crude reaction mixture is purified to isolate the individual components (typically using chromatography such as TLC, HPLC, SFC *etc.*), however multiple separations are often required to generate sufficiently pure compounds.^9^ Once the pure components have been isolated, the next challenge is to elucidate their structure. This generally requires either high-resolution mass spec (HRMS) or elemental analysis (EA) to understand the elemental composition, followed by NMR analysis to unambiguously assign the atomic connectivity.^10^–^13^

While this approach is amenable to a variety of synthetic platforms, there are several drawbacks regarding structural characterization. First, this is a very laborious process that requires many manual steps to obtain pure products for subsequent analyses. If the separation is challenging, this can often require additional purifications to be carried out, costing not only time but losing precious amounts of material to multiple separation passes. Additionally, it may be challenging to detect and isolate a sufficient quantity of small components of larger mixtures. Identifying these small abundance compounds may be crucial to understanding the reaction mechanism and can even lead to the development of new reaction methodologies. Finally, accurately quantifying products and reactants from a crude mixture can be challenging due to different ionization properties or UV responses of the various components. For all of these reasons, it is necessary to develop techniques that allow reaction yield and byproduct content to be determined directly on crude materials.

Molecular rotational resonance (MRR), also widely known in the literature as microwave or rotational spectroscopy, is a field with a long history within chemistry.^14^,^15^ This technique has historically been used in chemical physics because of its ability to provide insights into the electronic structure of gas phase molecules and weakly bound complexes, particularly in the study of intermolecular and intramolecular interactions.^16^–^19^ Recent advances in instrumentation have facilitated the measurement of broad-bandwidth spectra, enabling rapid characterization of mixtures, in a reasonable amount of time (minutes to hours),^20^–^22^ including for increasingly large, conformationally complex, and chiral compounds.^23^–^27^ Several recent studies have also demonstrated the capability for rotational spectroscopy to resolve numerous mixture components without purification.^24^,^28^–^30^ As a result, MRR has become suitable for characterization of compounds of industrial and pharmaceutical relevance.^31^

MRR identifies molecules through characterization of their pure rotational transitions in the gas phase, the energies of which are governed by their molecular rotational constants. These constants (*A*, *B*, and *C*) have an inverse proportional relationship with the molecule's three-dimensional moments of inertia and give rise to spectra with a multitude of highly resolved lines, as long as the compound has a permanent dipole moment. The permanent dipole moment magnitude and orientation directly affects the transition intensities, and quadrupolar nuclei (for example, ^14^N) also add hyperfine structure to the spectrum, which can be compared to calculations to provide additional structural information. Subtle differences in structure (including between regioisomers, diastereomers, and isotopologues) produce significant and calculable effects on the rotational constants, allowing for unambiguous confirmation of molecular identity. These parameters can be accurately calculated from molecular geometry, and have been used to benchmark computational methods for molecular structure.^32^ Additionally, techniques to elicit enantiomerically selective MRR spectra have recently been reported.^24^,^26^,^33^ The close relationship between spectra and calculations, along with the intrinsically high resolution of the technique, enables the identification of compounds directly within a mixture, without the need for pure reference standards. Hence, MRR is an ideal tool for the characterization of complex synthetic organic reactions.

In this paper, we demonstrate the ability of MRR to identify and quantify impurities in two different previously reported synthetic reactions. The first was presented in a recent study that reported the use of decatungstate photocatalysis to facilitate direct arylation of aliphatic C–H bonds to access complex and pharmaceutically relevant molecules.^34^ However, due to the variety and abundance of C–H bonds present in a given molecule, C–H arylation isomers often resulted. Herein, we report the application of MRR for the direct analysis of the photocatalytic arylation of cyclohexanone **1** with 5-bromo-2-(trifluoromethyl)pyridine **2** (Scheme 1a).

**Scheme 1:**
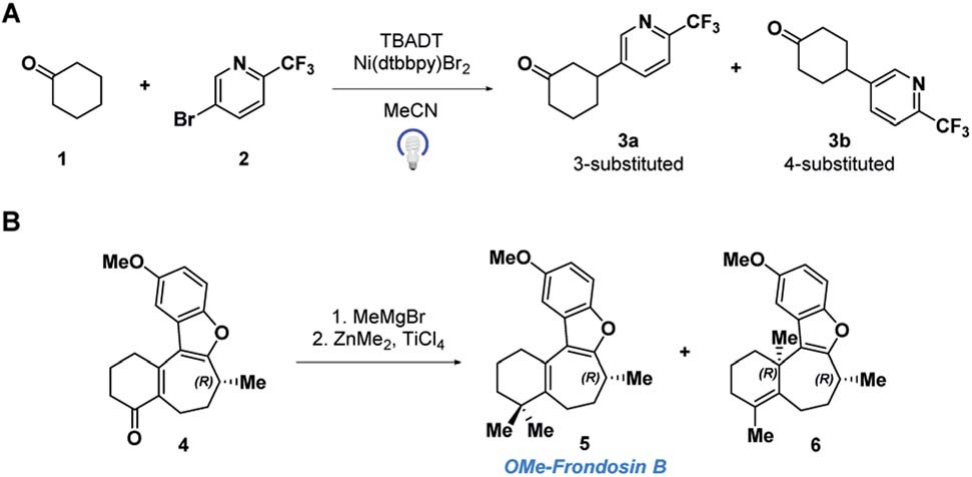
(A) Tetrabutylammonium decatungstate (TBADT) mediated C–H arylation, and (B) ketone methylation reactions analyzed directly with MRR.

The second reaction was part of an investigation into the absolute configuration of a natural product, frondosin B.^35^ As part of this study, previously reported syntheses of frondosin B were re-examined to determine the cause of a reversal in the sign of the optical rotation. While a stereochemical inversion was initially expected, it was discovered that near the end of the synthesis a two-step *gem*-dimethylation of ketone **4** produced a significant amount of regioisomeric impurity **6** (Scheme 1b). This impurity, as opposed to stereochemical inversion, accounted for the previously noted discrepancy.

Both of these reactions were characterized in previous studies by separation of the distinct products followed by structure elucidation, as described above. The purpose of the present study is to demonstrate the use of MRR spectroscopy to directly characterize the crude reaction mixtures and quantify their components with a single measurement. We successfully identified a number of components in both reactions, including both previously known and new impurities. All analyses were performed without the need for pure reference standards of the different products.

## Methods

MRR spectra of the two crude reaction mixtures were measured using a broadband chirped-pulse Fourier transform microwave spectrometer based at the University of Virginia, operating over the 2–8 GHz frequency range. The design of this spectrometer has been described elsewhere.^21^ In order to isolate the components in the gas phase and rotationally cool the samples for analysis, pulsed solenoid valves coupled to 0.9 mm pinhole nozzles are employed, which create a supersonic expansion of volatilized analytes in a carrier gas stream within a chamber maintained at high vacuum. This typically results in samples with rotational temperatures of approximately 1 K, cools out most vibrationally excited states, and also results in some conformational cooling (see further discussion below). The nozzles also include a heated reservoir for holding solid or liquid samples. The pulsed sample injection is configured to synchronize to a series of short (1–4 μs), high-power broadband chirped pulses. Between each pulse, the broadband microwave spectrum is recorded as a time-domain free induction decay (FID) signal, and Fourier transformed to yield the spectra presented below.

In these experiments, the samples (∼50 mg of unpurified oil) are dissolved in approximately 300 μL of dichloromethane, and divided between three identical reservoirs, which are configured to inject sample simultaneously to increase the measurement sensitivity. Neon (>99.999%) was used as the carrier gas. The sample reservoirs were first heated to 40 °C and pulsed to remove the dichloromethane solvent. Once this was completed, the reservoir temperatures were increased in steps of approximately 15 °C at a time, and the MRR spectrum was measured for approximately 5 minutes at each temperature step so that the presence of any impurities that are volatile at that temperature can be noted. The nozzles are operated at a 3 Hz repetition rate, with eight spectra measured on each injection pulse, and accumulated in the time domain to improve the sensitivity through signal averaging. The final measurement temperature, where the signals of the target compounds were optimized, was 165 °C for the cyclohexanone reaction, and 195 °C for the frondosin B mixture. In the cyclohexanone sample, volatile impurities were also observed at low temperature, which will be described in more detail below.

**Fig. 1:**
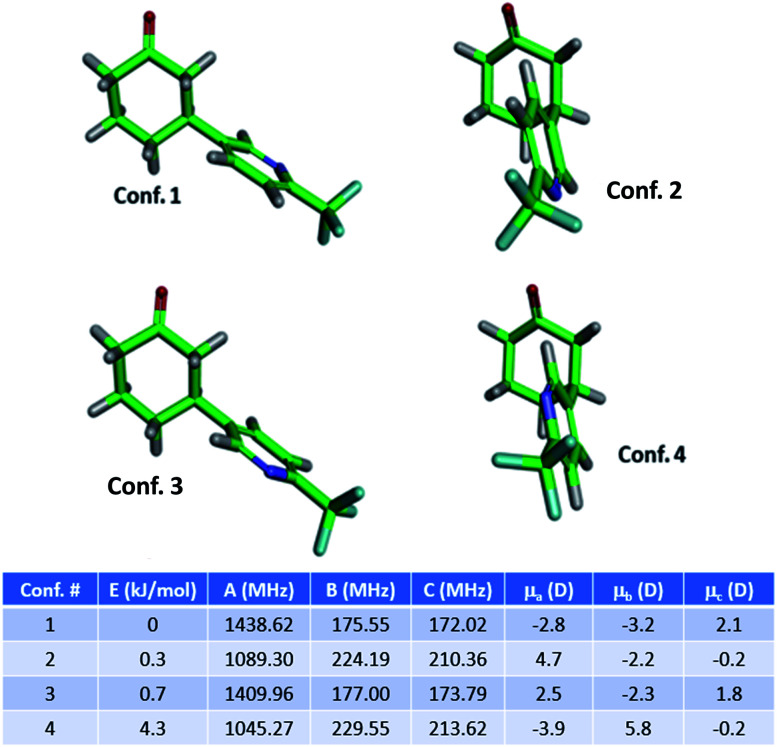
Structures of the four lowest energy conformers of the primary product **3a** (3-substituted) of the arylation reaction, and their calculated MRR parameters (rotational constants and dipole moment projections). Energies and rotational constants are calculated at a B3LYP-D3BJ/def2TZVP level of theory.

The MRR parameters of each compound of interest were also predicted computationally to facilitate component identification and quantification. First, a conformational search was performed on each compound to generate the possible minima, which is carried out using previously described methodology.^36^ These structures are then optimized using B3LYP calculations with the D3BJ dispersion correction as implemented in Gaussian 09.^37^–^39^ The D3BJ dispersion correction has been reported to improve the accuracy of the three-dimensional geometry, to which the MRR spectrum is extremely sensitive. In addition to observing substantial differences in the rotational constants between regioisomers, different conformational isomers of the same compound have distinct rotational spectra, and so it is important to include all low-energy conformers in the search. As an example, the conformers of **3a** (the major product of the arylation reaction) are presented in Fig. 1. In two of these conformers (**conf. 1** and **conf. 3**), the pyridine is in an equatorial position on the cyclohexanone ring, differing only by 180° rotation about the carbon-to-carbon bond between the two rings. Fig. 1 shows that despite this subtle difference in structure, the rotational constants are substantially different. The direction of the molecular dipole moment, presented through its projections along the three principal axes (*μ*_*a*_, *μ*_*b*_, and *μ*_*c*_), and the orientation of the ^14^N quadrupolar coupling constant (not shown here, but available in the ESI†), are also distinct between the two conformers, enabling their unambiguous identification in the sample. Likewise, the two axial conformers (**conf. 2** and **conf. 4**), can also be differentiated through their MRR parameters. The same computational method was carried out for the other species under consideration in both reactions, with full parameters available in the ESI.†

## Results and discussion

### Cyclohexanone arylation reaction

**Fig. 2:**
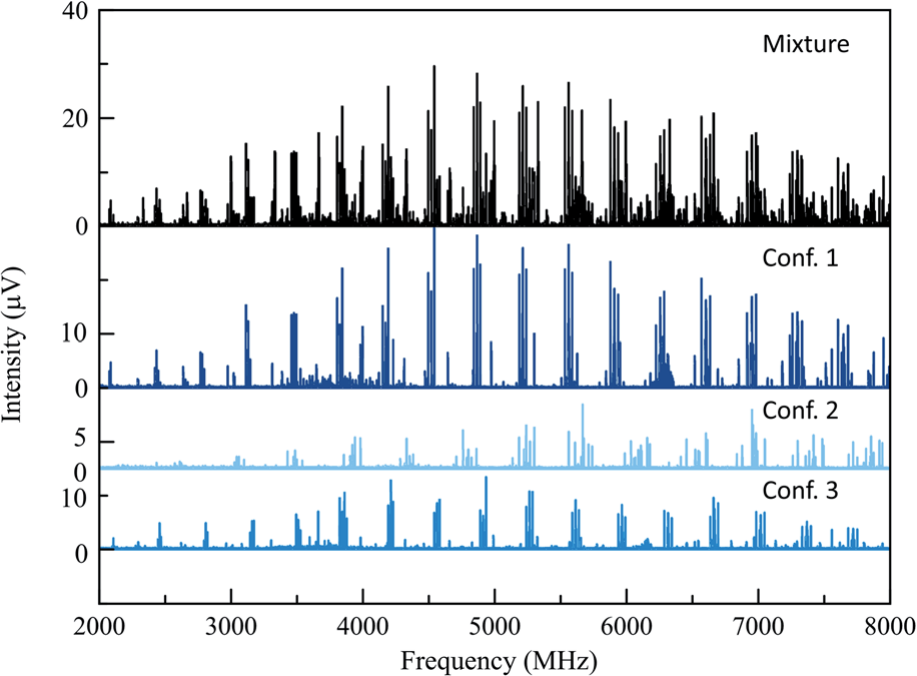
The top spectrum (in black) shows the full MRR spectrum of the cyclohexanone reaction mixture at a temperature of 165 °C. Beneath this are isolated MRR spectra of the three detected conformers of the major (3-substituted) product **3** in the arylation reaction.

The 165 °C broadband MRR spectrum of the crude arylation reaction mixture was measured over the course of 2.3 h (200 000 total spectral acquisitions). The spectrum is presented in the top panel of Fig. 2. The primary components in the sample (**3a** and **3b**) can be observed within a matter of seconds, while signal averaging enables the detection of minor components (1% or less). A minor regioisomer not indicated in the previous study, where arylation occurs at the 2-position, is identified. Each species is assigned by a fit to a rotational Hamiltonian with standard methods and software, using the theoretical rotational constants described above as a guide. Identifications are based on satisfactory agreement between experimental and computed rotational constants. Full fit results and procedures are available in the ESI.†

**Fig. 3:**
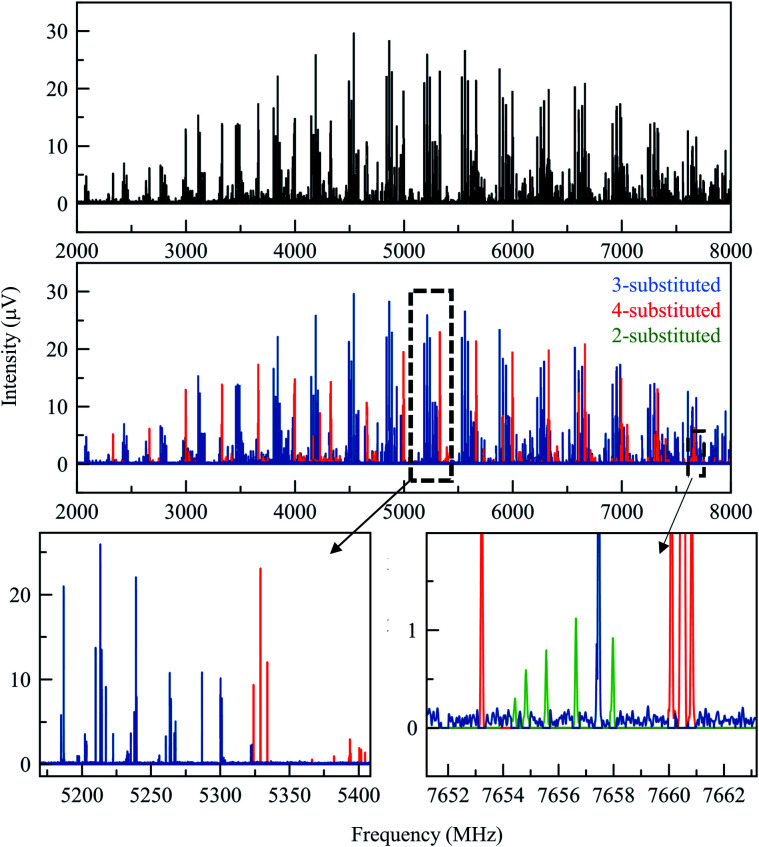
(Top) Broadband MRR spectrum of the arylation product mixture at 165 °C (see the text for full measurement description). The lower panels show the same spectrum, with the assigned contributions from each of the three product isomers colored to show their presence in the spectrum. (Different conformers of the same isomer are colored the same in this figure). The bottom row shows two expanded views to show that there are no spectral overlaps between the features of different species.

A total of three conformers of the primary 3-substituted product **3a** were identified in the MRR spectrum, along with two conformers of the 4-substituted isomer **3b**, and one of the 2-substituted isomer. Each conformer is fit separately in the spectrum. In Fig. 2, the full spectrum of the product mixture is shown in black, and the three observed conformers of **3a** are shown in different shades of blue in order to highlight their different contributions to the spectra. In the colored spectra, the spectral lines attributed to the indicated conformer have been kept in the spectrum while all of the other spectral lines have been cut for visual clarity. In Fig. 3, the full spectrum is again shown in black in the top panel. In the middle and lower panels, the transitions are color-coded based on which of the three regioisomers they have been assigned to. A portion of the spectrum of the minor 2-substituted isomer can be seen in the inset of the lower right corner of this figure.

Notably, the conformational population in pulsed supersonic expansion measurements does not reflect thermodynamic equilibrium at the sample vaporization temperature, as conformational cooling is typically observed. This effect has been explored in a number of previous studies.^40^–^42^ In order to quantify the total abundance of each isomer in the mixture, we sum the contributions of all of the identified conformers. The relative concentration of each conformer in the sample vapor was determined by simulating the MRR spectral pattern with the calculated molecular dipole moment components to predict the expected relative line intensities for each transition in the spectrum, using a rotational temperature of 1.5 K as this temperature best describes the spectral intensity pattern. The ratio between the experimental and simulated intensity therefore provides a relative measure of that component's abundance in the mixture. Averaging across all of the detected transitions of each conformer improves the quantitative accuracy. Once a scale factor is determined for each conformer of each species, the total abundance of each component can be calculated. Further information on the quantification procedure used here is available in the ESI.† This analysis did not account for any possible difference in vapor pressure between the regioisomers, which are expected to be small.

Table 1 shows the combined results of the analysis on the crude arylation sample. The ratio between isomers **3a** and **3b** were previously determined by HPLC is in excellent agreement with the MRR results. After the MRR results were obtained, the low-level presence of the 2-substituted isomer in this reaction mixture was confirmed by HPLC-MS. The estimated relative error for the quantitative results presented in this paper is about 10%, with the primary source being the calculation of the dipole moment of each species.

**Table 1:** Comparison of amounts of each substituted product of **3** determined by MRR and UPLC (note that the amount by UPLC represents area percent at 210 nm)

Compound	Amount (MRR)	Amount (UPLC)
**3a** (3-substituted)	57.0%	58.0%
**3b** (4-substituted)	42.7%	41.6%
2-substituted	0.3%	0.4%

**Fig. 4:**
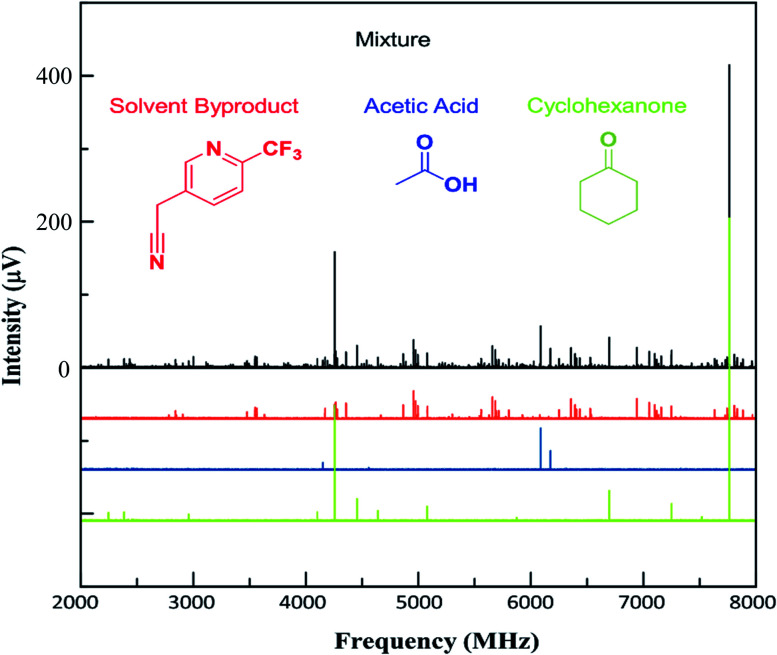
Lower-temperature (120–130 °C) spectrum of the arylation product mixture. The strongest line is due to the cyclohexanone reagent. Weaker lines are due to the solvent-derived byproduct. The arylation reaction products are also present at lower levels.

As noted above, this sample also contained additional impurities that were more volatile than the arylation products. While the current MRR measurement procedure does not reliably quantify these components (since they are measured at lower temperature), it is still notable that MRR can identify additional non-isomeric byproducts in mixtures as well as isomeric ones. Fig. 4 shows a spectrum that was measured across the temperature range 120–130 °C, where the vapor is dominated by lower molecular-weight impurities in the sample. Three primary components were identified in this spectrum. Two of these, cyclohexanone and acetic acid, had previously characterized MRR spectra^43^,^44^ and so were immediately identified using a spectral library. Cyclohexanone, in particular, is a starting material in the reaction, and was charged in excess, accounting for its presence in the mixture. The third byproduct identified was arylated acetonitrile (reaction solvent) with **2**. The structure was determined by first noting hyperfine structure in the impurity consistent with two ^14^N nuclei, and observing that acetonitrile is also a viable substrate for the arylation reaction. Electronic structure calculations were then performed on the proposed structure to confirm the molecular identification.

### Frondosin B intermediate reaction

**Fig. 5:**
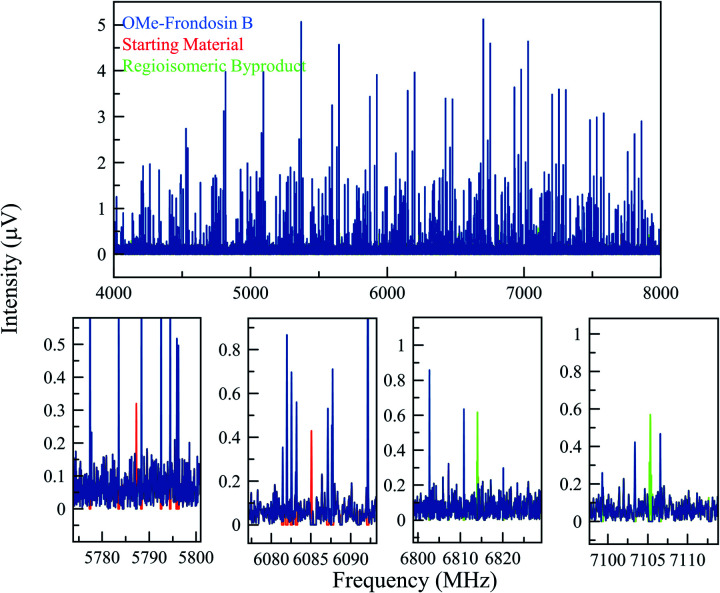
(Top) MRR spectrum of the OMe-frondosin B **5** reaction mixture (displayed from 4–8 GHz for clarity). The blue spectrum consists of the isolated spectra of four conformers of OMe-frondosin B (**5**), which make up all the strong lines observed. (Bottom) Expanded views of different regions of the spectrum to indicate some of the identified transitions of the regioisomeric impurity and starting material in the reaction. Because of the low concentration of these impurities, fewer lines are visible, but still can be matched unambiguously to a set of rotational constants that agrees with the calculations for these species.

The *gem*-dimethylation of the ketone **4** to form OMe-frondosin B (**5**) was measured for 295 000 signal averages (3.4 h). The resulting spectrum was then analyzed following the same procedure as for the first study. Fig. 5 presents the MRR spectrum of the crude reaction mixture. The spectrum is again dominated by several conformers of the primary product, with lower-abundance species appearing as weaker patterns in the spectrum. As was observed for the arylation reaction, the low-abundance species do not spectrally overlap with the primary mixture component.

The compounds in this sample did present additional conformational flexibility, due to the fused cyclohexene and cycloheptadiene rings that can obtain several energetically favorable conformations; these were previously described in greater detail.^35^ Additionally, the methyl ether group can adopt two configurations that are planar with the phenyl ring it is connected to. Crucially for the MRR analysis, one of these two configurations of OMe-frondosin B **5** results in a strong molecular dipole moment (>2 Debye), while the other has very low dipole moment (<0.3 Debye). The MRR signal response in the spectrometer used in this study is proportional to the square of the dipole moment, so the measurement sensitivity to half of the conformations is reduced by a factor of approximately 50 and are not detected. We assumed a conformational equilibrium at a temperature of 200 K (a best-fit based on the populations of the four observed conformers) and use this to correct for the populations expected for the low-dipole components. Under this model, only 17% of the OMe-frondosin B population is calculated to be in the low-polarity conformers, and so the precise value of the equilibrium temperature does not significantly affect the quantitative results presented below. This procedure is described in more detail in the ESI.†

**Fig. 6:**
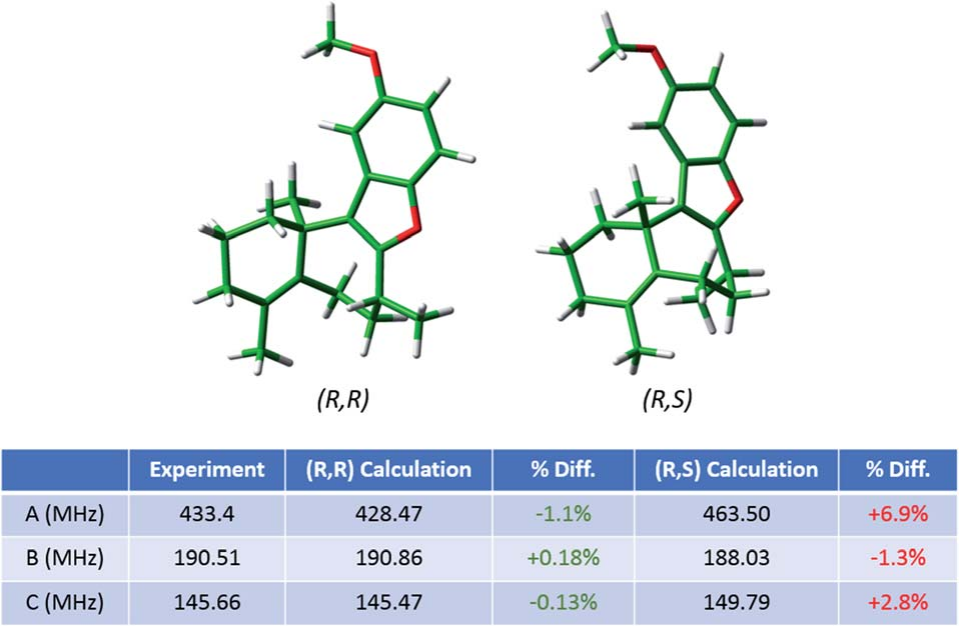
Calculated structures of the lowest-energy conformers of the regioisomeric byproduct in the frondosin B reaction (**6**) and its diastereomer. The table shows the experimental rotational constants assigned for this compound in the MRR crude reaction mixture analysis, and the percent differences between the experiment and each of the two calculations. The identified impurity is unambiguously assigned as the (*R*,*R*) diastereomer, consistent with the previous study.

As the regioisomeric byproduct **6** contains two chiral centers, in the previous study of this reaction^35^ the relative stereochemistry was determined on the basis of ^1^H ROESY 2-D NMR experiments on an isolated sample with comparison to chemical shifts predicted using DFT. Just as for regioisomers, MRR is extremely precise in distinguishing between diastereomers on the basis of their differences in moments of inertia. This is illustrated in Fig. 6, where the calculated minimum-energy conformers of compound **6** (*R*,*R*) and its diastereomer (*R*,*S*) are presented in comparison to the experimentally determined rotational constants for the impurity. The significant difference between the rotational constants of the two diastereomers, and the excellent agreement between the experimental constants and the calculation for the (*R*,*R*) diastereomer, demonstrates that MRR is able to unambiguously determine the stereochemistry of the impurity directly in the reaction mixture.

The final quantitative results for the OMe-frondosin B sample are presented in Table 2. Again we note that the agreement of MRR results with the previously reported chromatographic analysis (in this case, SFC) are in excellent agreement considering the measurement uncertainty of the two techniques.

**Table 2:** Comparison of amounts of each substituted product of in the synthesis of OMe-frondosin B **5** determined by MRR and SFC (note that the amount by SFC represents area percent at 210 nm)

Compound	Amount (MRR)	Amount (SFC)
OMe frondosin B (**5**)	91.2%	92.9%
Regioisomer (**6**)	6.8%	7.1%
Starting material (**4**)	2.0%	n.d.^*a*^

^*a*^The presence of the starting material was not noted on the SFC method that was used for analysis.

## Conclusions

We have demonstrated that MRR can be used for the direct characterization of crude reaction mixtures without reference standards. This technique was applied to two systems, both involving the identification and quantification of regioisomeric impurities in crude reaction mixtures: the photocatalytic C–H arylation of cyclohexanone, and the quantification of an isomeric impurity in an intermediate step in the synthesis of frondosin B. MRR streamlines the typical workflow of extensive purifications and structure elucidation techniques used for separation and confirmation of chemical structures. The quantitation results that were determined for the composition of both reaction mixtures are in good agreement with analytical results. Additionally, MRR can readily identify low-level impurities that would be challenging to separate and identify by alternative techniques. The ability to identify additional sites of activation in small quantities can be very important in a discovery chemistry setting where it could enable more rapid exploration of structure–activity relationships.

## Conflicts of interest

Authors J. L. N and B. H. P. have a financial interest in BrightSpec, Inc.

## Supplementary Material

Supplementary information
