# Supplementary material for: Direct regioisomer analysis of crude reaction mixtures via molecular rotational resonance (MRR) spectroscopy
Source: Chem Sci. 2020 Jun 8;11(24):6332–8. doi: 10.1039/d0sc01853h (PMC7472927; doi:10.1039/d0sc01853h)
Supplement: Supplementary file 1 [file SC-011-D0SC01853H-s001.pdf]

Electronic Supplementary Material (ESI) for Chemical Science.
This journal is © The Royal Society of Chemistry 2020

Direct Regioisomer Analysis of Crude Reaction Mixtures via Molecular Rotational Resonance (MRR) Spectroscopy: Electronic Supplementary Material (ESI)

Leo A. Joyce[a†], Danielle M. Schultz[a*], Edward C. Sherer[b], Justin L. Neill[c*], Reilly E. Sonstrom[d], and Brooks H. Pate[d]

[a]Department of Process Research & Development, Merck & Co., Inc., Rahway, NJ 07065, USA

[b]Department of Computational and Structural Chemistry, Merck & Co., Inc., Rahway, NJ 07065, USA

[c]BrightSpec, Inc., 770 Harris St., Suite 104b, Charlottesville, VA 22904, USA

[d]Department of Chemistry, University of Virginia, McCormick Road, Charlottesville, VA 22904, USA

†Current address: Arrowhead Pharmaceuticals, 502 S. Rosa Rd., Madison, WI 53719, USA, email: ljoyce@arrowheadpharma.com

Table of Contents

1) Computational Methods and Results

2) Molecular Rotational Resonance Results: Fit Parameters

3) Molecular Rotational Resonance Results: Quantitation of Isomeric Ratios

1) Computational Methods and Results

The methods for predicting molecular rotational resonance (MRR) parameters are similar to those described in a previous paper.[1] A molecular mechanics search algorithm is used to identify all possible minima on the potential energy surface. These searches were performed both using the Merck conformational sampling approach previously described,[2] and using the Compute*VOA* software (BioTools, Inc., Jupiter, FL) using the GMMX approach with MMFF94 force field. Each candidate conformer returned by these methods is then optimized using Gaussian 09.[3] Calculations are performed at a B3LYP-D3BJ/6-311++G(d,p) or B3LYP-D3BJ/def2TZVP level of theory. After all conformer structures are calculated, they are reordered and numbered based on their relative energies in the tables below.

Conformers in **bold** are those that are observed experimentally, while those in gray have low dipole moments and therefore are not expected to be observed with good sensitivity in MRR spectroscopy. The structures of conformers that are observed experimentally are depicted in the following section. In the table below, conformers up to 6 kJ mol^{-1} above the minimum energy conformer are presented.

a) Arylation Reaction

3-Substitued Arylation Product

Conformer #	A (MHz)	B (MHz)	C (MHz)	μ_a (Debye)	μ_b (Debye)	μ_c (Debye)	E (kJ/mol)
**1**	**1438.62**	**175.55**	**172.02**	**2.8**	**3.2**	**2.1**	**0**
**2**	**1089.30**	**224.19**	**210.36**	**4.7**	**2.2**	**0.2**	**0.3**
**3**	**1409.96**	**177.01**	**173.79**	**2.5**	**2.3**	**1.8**	**0.7**
4	1045.27	229.55	213.62	3.9	5.8	0.2	4.3

Calculations performed at a B3LYP-D3BJ/def2TZVP level of theory.

4-Substitued Arylation Product

Conformer #	A (MHz)	B (MHz)	C (MHz)	μ_a (Debye)	μ_b (Debye)	μ_c (Debye)	E (kJ/mol)
**1**	**1653.24**	**166.76**	**166.17**	**2.0**	**0.2**	**0.0**	**0**
**2**	**1584.74**	**169.08**	**167.74**	**1.6**	**4.2**	**0.0**	**1.6**
3	1276.78	196.42	189.14	1.9	0.9	0.0	13.2

4	1198.24	202.77	193.07	0.9	4.6	0.0	14.2

Calculations performed at a B3LYP-D3BJ/def2TZVP level of theory.

2-Substitued Arylation Product

Conformer #	A (MHz)	B (MHz)	C (MHz)	μ_a (Debye)	μ_b (Debye)	μ_c (Debye)	E (kJ/mol)
**1**	**1407.46**	**194.17**	**188.18**	**6.3**	**1.9**	**1.2**	**0**
2	1376.13	196.28	191.36	6.0	3.5	2.1	2.7
3	1081.49	226.43	211.02	3.7	1.0	0.1	5.9
4	1088.20	225.74	211.14	3.9	4.7	0.1	8.1

Calculations performed at a B3LYP-D3BJ/def2TZVP level of theory.

Solvent Byproduct

Conformer #	A (MHz)	B (MHz)	C (MHz)	μ_a (Debye)	μ_b (Debye)	μ_c (Debye)	E (kJ/mol)
**1**	**2340.98**	**370.15**	**339.43**	**1.6**	**1.6**	**0.0**	**0**
2	2206.24	378.73	343.52	1.2	5.5	0.0	3.2

Calculations performed at a B3LYP-D3BJ/def2TZVP level of theory.

a) Frondosin B Reaction

OMe-Frondosin B

Conformer #	A (MHz)	B (MHz)	C (MHz)	μ_a (Debye)	μ_b (Debye)	μ_c (Debye)	E (kJ/mol)
**1**	**377.93**	**191.44**	**138.4**	**2.0**	**1.2**	**0.2**	**0**
**2**	**387.48**	**188.08**	**137.83**	**1.9**	**1.3**	**0.2**	**0.8**
**3**	**377.20**	**193.24**	**136.99**	**2.0**	**1.2**	**0.1**	**2.3**
4	406.11	174.66	132.60	0.1	0.2	0.0	2.5
**5**	**421.27**	**183.85**	**142.23**	**1.9**	**1.3**	**0.1**	**2.7**
6	417.40	172.08	132.32	0.0	0.2	0.0	3.2
7	385.25	187.26	138.55	2.0	1.4	0.2	4.7
8	405.36	176.22	131.31	0.0	0.2	0.1	4.8
9	456.11	168.31	136.09	0.0	0.2	0.0	5.2
10	417.29	183.47	143.28	2.0	1.4	0.2	5.2
11	380.15	191.92	138.32	2.0	1.1	0.1	5.7

Calculations performed at a B3LYP-D3BJ/6-311++G(d,p) level of theory.

Regioisomeric Byproduct (R,R)

Conformer #	A (MHz)	B (MHz)	C (MHz)	μ_a (Debye)	μ_b (Debye)	μ_c (Debye)	E (kJ/mol)

**1**	**428.44**	**190.96**	**145.52**	**1.9**	**1.7**	**0.1**	**0**
2	448.75	190.43	152.60	1.7	1.6	0.2	1.7
3	462.36	175.77	139.60	0.3	0.0	0.3	2.5
4	483.14	176.13	146.34	0.2	0.2	0.2	4.1

Calculations performed at a B3LYP-D3BJ/6-311++G(d,p) level of theory.

Diastereomer of Regioisomeric Byproduct (R,S)

Conformer #	A (MHz)	B (MHz)	C (MHz)	μ_a (Debye)	μ_b (Debye)	μ_c (Debye)	E (kJ/mol)
1	463.50	188.03	149.79	1.9	1.7	0.2	0
2	502.94	172.99	143.39	0.3	0.0	0.2	2.4

Calculations performed at a B3LYP-D3BJ/6-311++G(d,p) level of theory.

Starting Material

Conformer #	A (MHz)	B (MHz)	C (MHz)	μ_a (Debye)	μ_b (Debye)	μ_c (Debye)	E (kJ/mol)
**1**	**404.90**	**213.89**	**148.87**	**2.6**	**0.6**	**1.6**	**0**
2	437.18	194.23	142.71	4.5	0.8	1.4	1.9
3	405.21	215.97	147.24	2.7	0.7	1.2	2.1
4	437.36	196.15	141.14	4.6	0.7	1.3	3.9
5	418.53	208.22	148.19	2.6	1.1	1.7	5.1
6	413.91	208.28	149.12	2.6	1.1	1.7	5.5
7	409.80	213.47	148.84	2.5	0.6	1.6	5.8

Calculations performed at a B3LYP-D3BJ/6-311++G(d,p) level of theory.

2) Molecular Rotational Resonance Results: Fit Parameters

General Notes

Each assigned compound in the molecular rotational resonance spectrum was fit to a rotational Hamiltonian using Pickett's SPFIT/SPCAT program,[4] with parameter standard errors derived using Kisiel's PIFORM program.[5]

For the weaker compounds (the regioisomeric byproduct and starting material) detected in the frondosin B reaction, the uncertainty on the *A* and *B* rotational constants is higher than in the other fits due to a strong correlation in the fit resulting from only observing the strongest lines of the compound. The identification of the compounds, and their correspondence to the assigned structures, is nevertheless secure.

Arylation Reaction

3-Substituted Arylation Product (3a)

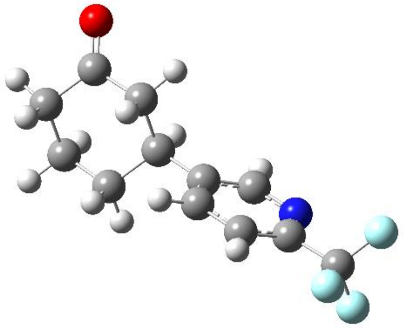

	Conformer 1		
	Experiment	Calculation	% Diff.
A (MHz)	1435.7522(3)	1438.62	+0.20%
B (MHz)	175.54050(3)	175.54	0.00%
C (MHz)	172.05277(3)	172.02	-0.02%
(^{14}N) 1.5χ_{aa} (MHz)[1]	-0.93(3)	-1.24	
(^{14}N) 0.25($\chi_{bb}-\chi_{cc}$) (MHz)	1.391(3)	1.56	
μ_a (D)		2.8	
μ_b (D)		3.2	
μ_c (D)		2.1	
N_{lines}[2]	206		
σ (kHz)[3]	9.5		

[1]Nuclear quadrupole coupling constants.

[2]Number of independent lines in the fit.

[3]Root-mean-square error between experimental line frequencies and simulated using experimental parameters.

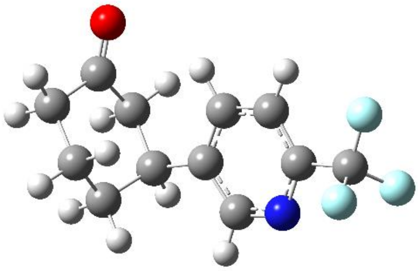

	Conformer 2		
	**Experiment**	**Calculation**	**% Diff.**
A (MHz)	1086.3188(10)	1089.30	+0.27%
B (MHz)	224.91898(10)	224.19	-0.32%
C (MHz)	210.92085(7)	210.36	-0.27%
(^{14}N) 1.5χ_{aa} (MHz)	-2.27(14)	-2.65	
(^{14}N) 0.25($\chi_{bb}-\chi_{cc}$) (MHz)	-1.279(7)	-1.44	
μ_a (D)		4.7	
μ_b (D)		2.2	
μ_c (D)		0.2	
N_{lines}	59		
σ (kHz)	9.1		

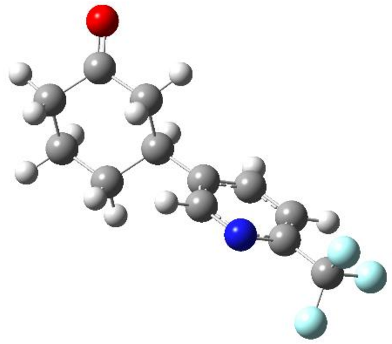

	Conformer 3		
	**Experiment**	**Calculation**	**% Diff.**
A (MHz)	1406.7166(12)	1409.96	+0.23%
B (MHz)	177.04336(14)	177.01	-0.02%
C (MHz)	173.89213(13)	173.79	-0.06%
(^{14}N) 1.5χ_{aa} (MHz)	0.41(19)	0.28	
(^{14}N) 0.25($\chi_{bb}-\chi_{cc}$) (MHz)	1.573(11)	1.76	
μ_a (D)		2.5	
μ_b (D)		2.4	
μ_c (D)		1.8	
N_{lines}	161		
σ (kHz)	9.0		

4-Substituted Arylation Product (3b)

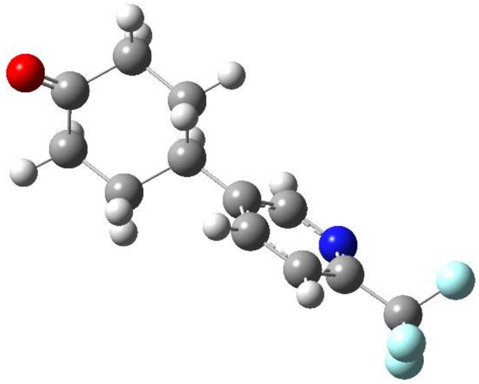

	Conformer 1		
	**Experiment**	**Calculation**	**% Diff.**
A (MHz)	1641.66(23)	1653.24	+0.71%
B (MHz)	166.84461(10)	166.76	-0.05%
C (MHz)	166.22100(10)	166.17	-0.03%
(^{14}N) 1.5χ_{aa} (MHz)	-1.56(4)	-1.71	
(^{14}N) 0.25($\chi_{bb}-\chi_{cc}$) (MHz)	-1.59(22)	-1.65	
μ_a (D)		2.0	
μ_b (D)		0.2	
μ_c (D)		0.0	
N_{lines}	72		
σ (kHz)	9.2		

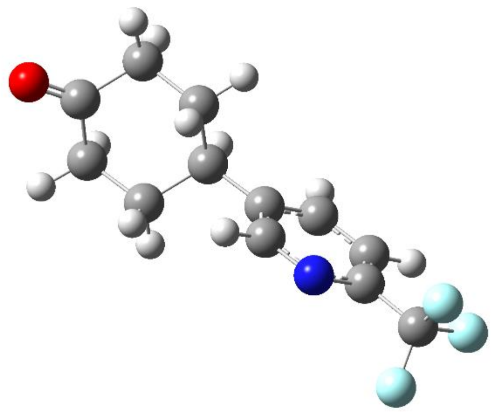

	Conformer 2		
	**Experiment**	**Calculation**	**% Diff.**
A (MHz)	1578.9148(3)	1584.74	+0.37%
B (MHz)	169.25353(3)	169.08	-0.10%
C (MHz)	167.84919(3)	167.74	-0.06%
(^{14}N) 1.5χ_{aa} (MHz)	0.95(4)	0.64	
(^{14}N) 0.25($\chi_{bb}-\chi_{cc}$) (MHz)	-1.841(3)	-2.05	
μ_a (D)		1.6	
μ_b (D)		4.2	
μ_c (D)		0.0	
N_{lines}	123		
σ (kHz)	7.4		

2-Substituted Arylation Product

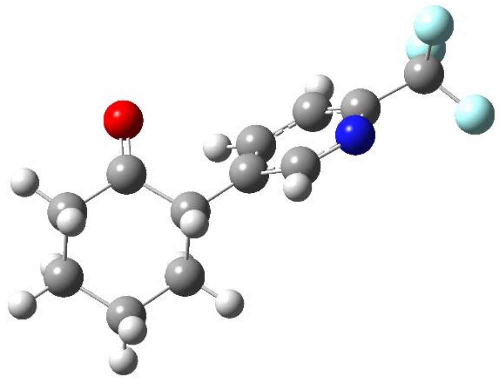

	Conformer 1		
	**Experiment**	**Calculation**	**% Diff.**
A (MHz)	1403.6(7)	1407.46	+0.27%
B (MHz)	194.3298(5)	194.17	-0.08%
C (MHz)	188.3470(5)	188.18	-0.09%
(^{14}N) $1.5\chi_{aa}$ (MHz)	--[1]	-1.44	
(^{14}N) $0.25(\chi_{bb}-\chi_{cc})$ (MHz)	--[1]	0.81	
μ_a (D)		6.3	
μ_b (D)		1.9	
μ_c (D)		1.2	
N_{lines}	85		
σ (kHz)	8.6		

[1]For this isomer, quadrupole hyperfine structure was not modeled as not enough lines with visible splitting were detected.

Solvent Adduct Impurity

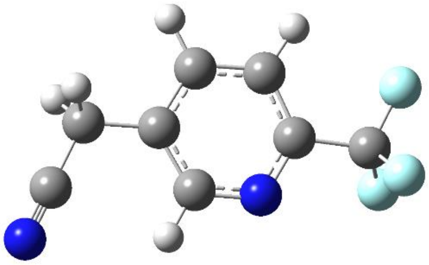

	Conformer 1		
	**Experiment**	**Calculation**	**% Diff.**
A (MHz)	2330.8998(13)	2340.98	0.43%
B (MHz)	371.02923(29)	370.15	-0.24%
C (MHz)	340.12728(26)	339.43	-0.20%
($^{14}N_{pyridyl}$) $1.5\chi_{aa}$ (MHz)	-2.08	-2.32	
($^{14}N_{pyridyl}$) $0.25(\chi_{bb}-\chi_{cc})$ (MHz)	-1.37	-1.55	
($^{14}N_{nitrile}$) $1.5\chi_{aa}$ (MHz)	-0.27	-0.41	
($^{14}N_{nitrile}$) $0.25(\chi_{bb}-\chi_{cc})$ (MHz)	-0.94	-1.07	
μ_a (D)		1.6	
μ_b (D)		1.6	
μ_c (D)		0.0	
N_{lines}		218	
σ (kHz)		6.4	

b) Frondosin B Reaction

OMe-Frondosin B (5)

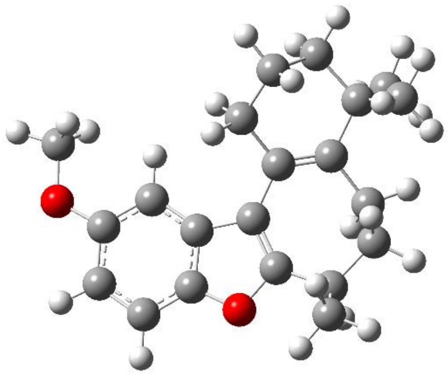

	Conformer 1		
	**Experiment**	**Calculation**	**% Diff.**
A (MHz)	378.51738(11)	377.93	-0.16%
B (MHz)	191.332581(27)	191.44	+0.06%
C (MHz)	138.348877(21)	138.4	+0.04%
μ_a (D)		2.0	
μ_b (D)		1.2	
μ_c (D)		0.2	
N_{lines}	358		
σ (kHz)	12.4		

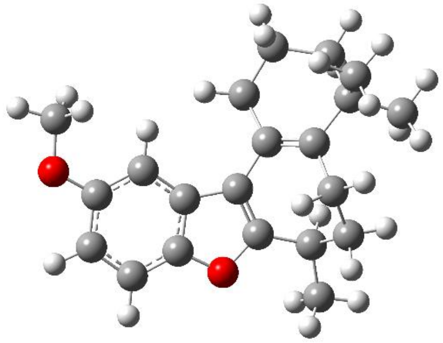

	Conformer 2		
	**Experiment**	**Calculation**	**% Diff.**
A (MHz)	389.71430(22)	387.48	-0.57%
B (MHz)	187.59687(5)	188.08	+0.26%
C (MHz)	137.99666(3)	137.83	-0.12%
μ_a (D)		1.8	
μ_b (D)		1.3	
μ_c (D)		0.1	
N_{lines}	299		
σ (kHz)	13.7		

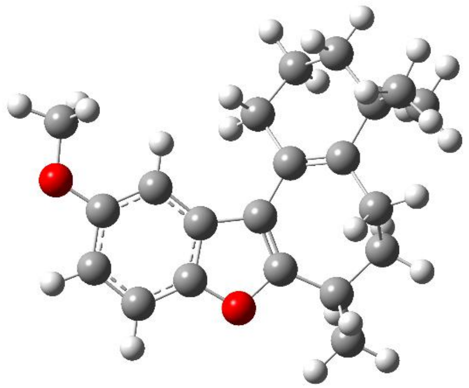

	Conformer 3		
	**Experiment**	**Calculation**	**% Diff.**
A (MHz)	378.43673(17)	377.20	-0.33%
B (MHz)	192.91255(5)	193.24	+0.17%
C (MHz)	137.18444(3)	136.99	-0.14%
μ_a (D)		2.0	
μ_b (D)		1.2	
μ_c (D)		0.1	
N_{lines}	191		
σ (kHz)	14.6		

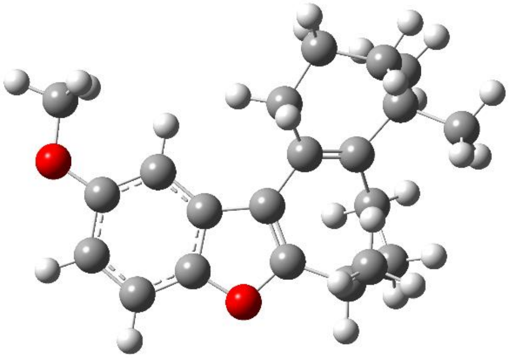

	Conformer 5		
	**Experiment**	**Calculation**	**% Diff.**
A (MHz)	421.878(3)	421.27	-0.14%
B (MHz)	183.7644(3)	183.85	+0.05%
C (MHz)	142.36831(9)	142.23	-0.09%
μ_a (D)		1.9	
μ_b (D)		1.3	
μ_c (D)		0.1	
N_{lines}	47		
σ (kHz)	13.9		

Regioisomeric Byproduct (6)

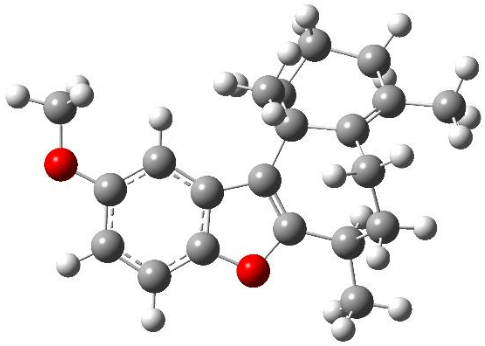

	Conformer 1		
	**Experiment**	**Calculation**	**% Diff.**
A (MHz)	433.4(4)	428.47	-1.14%
B (MHz)	190.51(7)	190.86	+0.18%
C (MHz)	145.65970(17)	145.47	-0.13%
μ_a (D)		1.9	
μ_b (D)		1.7	
μ_c (D)		0.1	
E (hartree)		-966.682763	
N_{lines}	21		
σ (kHz)	13.6		

Starting Material (4)

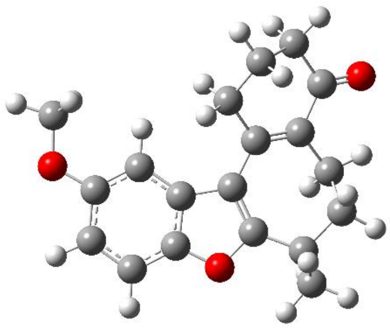

	Conformer 1		
	**Experiment**	**Calculation**	**% Diff.**
A (MHz)	404.9(4)	404.90	+0.01%
B (MHz)	213.90(11)	213.89	-0.01%
C (MHz)	148.89995(13)	148.87	+0.01%
μ_a (D)		2.6	
μ_b (D)		0.6	
μ_c (D)		1.6	
E (hartree)		-962.075599	
N_{lines}	20		
σ (kHz)	7.3		

3) Molecular Rotational Resonance Results: Quantitation of Isomeric Ratios

After each species assigned in the MRR spectrum is identified to a computed structure, the relative abundance of each is determined. This is done by simulating the spectrum using the experimental rotational constants, with *ab initio* dipole moments used to predict the signal intensity. These intensities are given in units of nm^2MHz. For each identified line in the spectrum, the ratio of the observed intensity (in millivolts, or mV) to the calculated intensity is calculated. The total scale factor for that structure is then determined by the mean ratio of the experimental to simulated line intensity across all of the assigned lines for that component.

In this analysis, weak-pulse scaling (that is, signal intensity proportional to the square of the transition moment) is assumed, which we validated experimentally by varying the microwave excitation pulse length and confirming that no species were excited past the optimal excitation condition. The relative error on the scale factor for each compound is estimated at 10%, which is dominated by the error in the calculated dipole moment.

For the regioisomeric impurity in the frondosin B reaction, all observed transitions have a triplet splitting pattern, which we have not fully modeled in this analysis but believe to be due to internal rotation. We have summed the intensity of each triplet for this analysis. For OMe-frondosin B, two species are observed that are assigned as vibrationally excited states of the lowest-energy conformer (based on having very similar rotational constants, within 1%). The contributions of these vibrational satellites are added to that of the main conformer, assuming the same dipole moment as the main conformer.

Arylation Reaction

Species	Best-Fit Scale Factor	Abundance (%)
3-Substituted	Total: 42.4	57.0%
Conformer 1	24.9	
Conformer 2	3.3	
Conformer 3	14.2	
4-Substituted	Total: 31.8	42.7%
Conformer 1	26.9	
Conformer 2	4.9	
2-Substituted	0.2	0.3%

Frondosin B Reaction

Species	Best-Fit Scale Factor	Abundance (%)
OMe-Frondosin B	Total: 26.9	91.2%
Conformer 1	9.7	
Conformer 2	5.8	
Conformer 3	3.2	
Conformer 5	1.6	
Assumed Population in Nonpolar Conformers (See below)	4.6	
Regioisomer Impurity	2.0	6.8%
Starting Material	0.6	2.0%

For the frondosin B reaction, we apply a correction for conformers of OMe-frondosin B that are not observed due to low polarity. For this analysis, we assume a conformational equilibrium temperature of 200 K. This is based on the observed populations of the four conformers of OMe-frondosin B that are observed in this analysis. In pulsed-jet MRR spectrometers such as the one used in this study, conformational cooling from the initial sample temperature (in this case, 468 K) is almost always observed, with the exact cooling depending on the energy barriers between conformers. For OMe-frondosin B, the most significant nonpolar conformers (4 and 6) require a

180-degree rotation of the methoxy group, which has a significant barrier (8 kJ mol^{-1}, calculated using Gaussian 09 at a B3LYP/6-311g level of theory). Therefore, we believe that these conformers likely still have substantial population in the pulsed jet.

In the figure below, the relative populations of the OMe-frondosin B conformers are presented, along with simulated populations assuming equilibrium temperatures of 150 K, 200 K, and 250 K. At 200 K, 17% of the OMe-frondosin B population is estimated to be in low-dipole moment conformers, as opposed to 10% at 150 K and 23% at 250 K. However, this is still a fairly minor correction to the final quantitative results, as the table below shows.

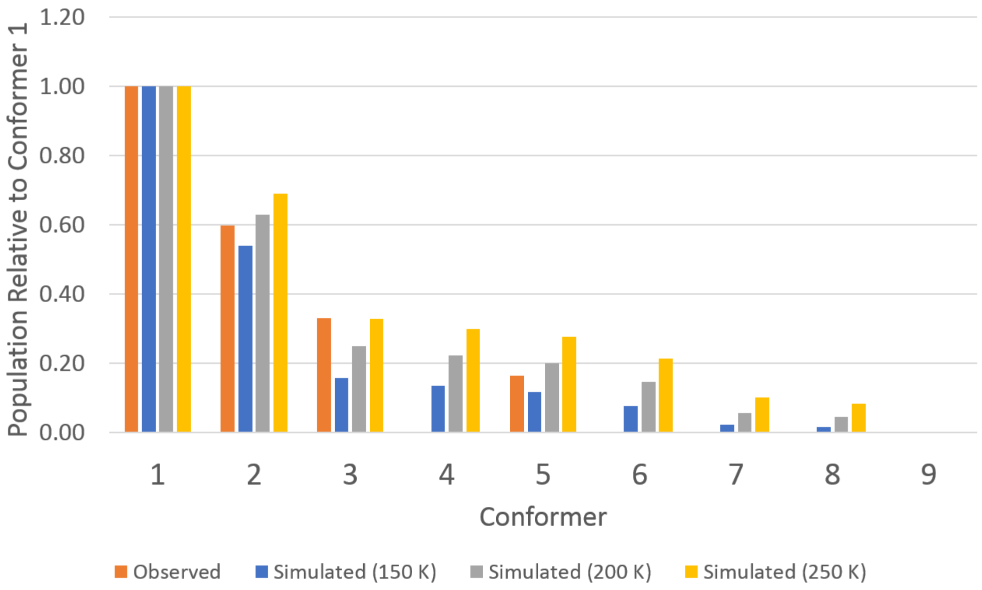

Assumed Conformational Equilibrium Temperature	150 K	200 K (best agreement with observed conformer populations)	250 K
OMe-frondosin B (**5**)	90.6%	91.2%	91.9%
Regioisomer impurity (**6**)	7.3%	6.8%	6.3%
Starting material (**4**)	2.1%	2.0%	1.8%

4) Chromatography Results: Quantitation of Isomeric Ratios

For the arylation reaction, isomeric ratios were determined by UPLC analysis of the crude reaction mixture (see below). The ratios were calculated by integration of the UV signal at 210 nm for each of the three isomer peaks. Chromatography conditions are shown below:

Instrument: Waters Acquity SQD-MS

Column: BEH C18 (150x2.1mm, 1.8um)

Mobile Phase A: 2 mM NH_4HCO_2 pH 3.5 in water

Mobile Phase B: 2 mM NH_4HCO_2 pH 3.5 in 90% MeCN/10% water

Gradient:

Time (min)	% Mobile Phase B
0	5%
4	95%
6	95%

Temperature: 50°C

Flow rate: 0.4 mL/min

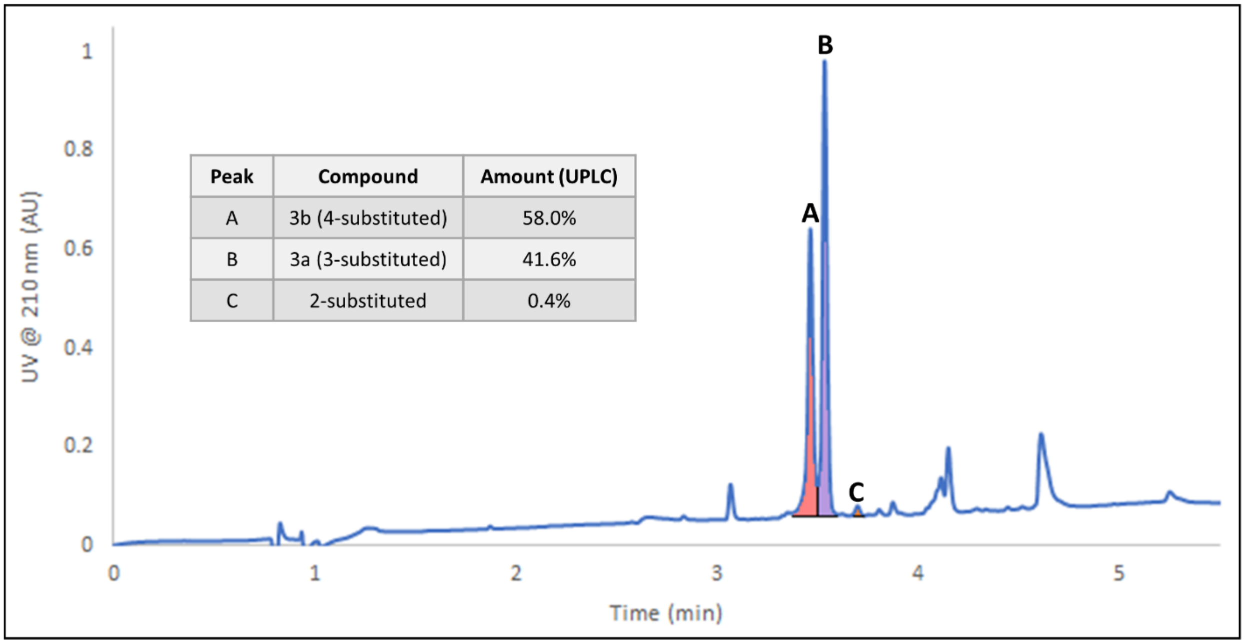

Peak	Compound	Amount (UPLC)
A	3b (4-substituted)	58.0%
B	3a (3-substituted)	41.6%
C	2-substituted	0.4%

For the frondosin B reaction, isomeric ratios were determined by SFC analysis of the crude reaction mixture. This data has been reported previously.[1]

References

1. L.A. Joyce, C.C. Nawrat, E.C. Sherer, M. Biba, A. Brunskill, G.E. Martin, R.D. Cohen, and I.W. Davis, *Chem. Sci.* 2018, **9**, 415.

2. E.C. Sherer, C.H. Lee, J. Shpungin, J.F. Cuff, C. Da, R. Ball, R. Bach, A. Crespo, X. Gong, and C.J. Welch, *J. Med. Chem.* 2014, **57,** 477.

3. Gaussian 09, Revision D.01, M. J. Frisch, G. W. Trucks, H. B. Schlegel, G. E. Scuseria, M. A. Robb, J. R. Cheeseman, G. Scalmani, V. Barone, G. A. Petersson, H. Nakatsuji, X. Li, M. Caricato, A. Marenich, J. Bloino, B. G. Janesko, R. Gomperts, B. Mennucci, H. P. Hratchian, J. V. Ortiz, A. F. Izmaylov, J. L. Sonnenberg, D. Williams-Young, F. Ding, F. Lipparini, F. Egidi, J. Goings, B. Peng, A. Petrone, T. Henderson, D. Ranasinghe, V. G. Zakrzewski, J. Gao, N. Rega, G. Zheng, W. Liang, M. Hada, M. Ehara, K. Toyota, R. Fukuda, J. Hasegawa, M. Ishida, T. Nakajima, Y. Honda, O. Kitao, H. Nakai, T. Vreven, K. Throssell, J. A. Montgomery, Jr., J. E. Peralta, F. Ogliaro, M. Bearpark, J. J. Heyd, E. Brothers, K. N. Kudin, V. N. Staroverov, T. Keith, R. Kobayashi, J. Normand, K. Raghavachari, A. Rendell, J. C. Burant, S. S. Iyengar, J. Tomasi, M. Cossi, J. M. Millam, M. Klene, C. Adamo, R. Cammi, J. W. Ochterski, R. L. Martin, K. Morokuma, O. Farkas, J. B. Foresman, and D. J. Fox, Gaussian, Inc., Wallingford CT, 2016.

4. H.M. Pickett, *J. Mol. Spectrosc.* 1991, **148**, 371. Available on the Web at https://spec.jpl.nasa.gov/.

5. Available on the Web at http://info.ifpan.edu.pl/~kisiel/prospe.htm.
